# Species-specific transcriptomic changes upon respiratory syncytial virus infection in cotton rats

**DOI:** 10.1038/s41598-022-19810-4

**Published:** 2022-10-04

**Authors:** Britton A. Strickland, Seesandra V. Rajagopala, Arash Kamali, Meghan H. Shilts, Suman B. Pakala, Marina S. Boukhvalova, Shibu Yooseph, Jorge C. G. Blanco, Suman R. Das

**Affiliations:** 1 Department of Pathology Microbiology and Immunology, Vanderbilt University Medical Center, Nashville, TN USA; 2 Division of Infectious Diseases, Department of Medicine, Vanderbilt University Medical Center, 1211 21st Avenue South, S2108 Medical Center North, Nashville, TN 37232 USA; 3 Sigmovir Biosystems Inc., 9610 Medical Center Drive, Suite 100, Rockville, MD 20850 USA; 4 Department of Computer Science, Genomics and Bioinformatics Cluster, University of Central Florida, Orlando, FL USA

**Keywords:** Viral pathogenesis, Virus-host interactions

## Abstract

The cotton rat (*Sigmodon*) is the gold standard pre-clinical small animal model for respiratory viral pathogens, especially for respiratory syncytial virus (RSV). However, without a reference genome or a published transcriptome, studies requiring gene expression analysis in cotton rats are severely limited. The aims of this study were to generate a comprehensive transcriptome from multiple tissues of two species of cotton rats that are commonly used as animal models (*Sigmodon fulviventer* and *Sigmodon hispidus),* and to compare and contrast gene expression changes and immune responses to RSV infection between the two species. Transcriptomes were assembled from lung, spleen, kidney, heart, and intestines for each species with a contig N50 > 1600. Annotation of contigs generated nearly 120,000 gene annotations for each species. The transcriptomes of *S. fulviventer* and *S. hispidus* were then used to assess immune response to RSV infection. We identified 238 unique genes that are significantly differentially expressed, including several genes implicated in RSV infection (e.g., *Mx2, I27L2*, *LY6E*, *Viperin*, *Keratin 6A, ISG15, CXCL10, CXCL11, IRF9*) as well as novel genes that have not previously described in RSV research (*LG3BP, SYWC, ABEC1, IIGP1, CREB1*). This study presents two comprehensive transcriptome references as resources for future gene expression analysis studies in the cotton rat model, as well as provides gene sequences for mechanistic characterization of molecular pathways. Overall, our results provide generalizable insights into the effect of host genetics on host-virus interactions, as well as identify new host therapeutic targets for RSV treatment and prevention.

## Introduction

Respiratory syncytial virus (RSV) is the leading cause of lower respiratory tract infection (LRTI) in children below the age of two years, as well as in immunocompromised individuals and the elderly, resulting in 33 million LRTIs, 3.2 million hospital admissions, and nearly 120,000 deaths worldwide each year^1^. There is currently no approved RSV vaccine and only one preventative monoclonal antibody (Palivizumab), with use limited to high-risk children due to costs^2,3^. As 93% of RSV LRTI cases and 99% of RSV mortality occurs in developing countries, the need for an effective vaccine and low-cost preventatives is critical^1^. The failure of the formalin-inactivated RSV vaccine in the 1960s, which induced enhanced disease in vaccinees upon encounter with the virus, hampered the development of new RSV vaccines for decades^4–6^. However, recently there is a renewed effort to develop RSV preventatives, with 14 vaccine candidates and alternative anti-viral strategies against RSV (recombinant antibodies^7^, nanobodies^8^, small molecule inhibitors and analogs^9^) that are at various stages of development^10^. This highlights the critical need for an appropriate pre-clinical model for vaccine and drug development against RSV.

The cotton rat (genus *Sigmodon*) is considered the “gold standard” animal model for RSV infection compared to mice and other animals because it is 100-fold more permissive than the majority of laboratory mice to RSV infection and RSV infects both its upper and lower respiratory tracts similar to humans^11–13^. Cotton rats have also accurately predicted efficacy of the two FDA-approved RSV therapeutics (RespiGam®, Palivizumab®)^14–17^. In addition to RSV, cotton rats have been used to study other human respiratory viruses of significance, i.e., influenza A virus^18,19^, parainfluenza virus^20,21^, measles^22^, human metapneumovirus^23^, enterovirus D68^24^, and human rhinovirus^25^, due to the broad susceptibility and comparable human disease features^26^. Unfortunately, studies comparing transcriptomic changes in cotton rats have been limited due to the lack of publicly available reference genome for any cotton rat species. Previously, we published a lung tissue transcriptome induced by RSV in *S. hispidus*^27^. However, in that study we had only analyzed expression in one tissue type, and it was limited to only one cotton rat species (*S. hispidus*). As prior studies show, both *S. fulviventer-* and *S. hispidus*-specific differ in disease severity to viral pathogens (i.e., parainfluenza virus^28^, HIV^29^) and microbiome community structure^30^. The main objectives of this study were to develop comprehensive transcriptomes for both species and to compare and contrast gene expression changes upon RSV infection.

To this end, we sequenced total RNA of multiple tissues (lung, spleen, heart, kidney, colon) from healthy animals and generated a comprehensive transcriptome using de novo assembly with functional annotation for two species of cotton rats (*S. fulviventer* and *S. hispidus*). We then infected each species of cotton rat with RSV A/Long strain alongside uninfected controls and used our transcriptome references to determine differentially expressed genes after RSV infection.

## Results

### RNA extraction and transcriptome sequencing

Total RNA was extracted from sections of spleen, heart, kidney, lung, and colon harvested from 4–6-week-old healthy *S. fulviventer* and *S. hispidus* for comprehensive transcriptome assembly (supplementary table 1). The RNA-seq libraries were sequenced to a depth of 50 million paired-end 2 × 150 per sample. After sequencing read QC, we pooled data from all healthy tissues (n = 2 per species, lung n = 4 per species) to generate 456 million paired-end reads for *S. fulviventer* (GC content 54.9%) and 465 million paired-end for *S. hispidus* (GC content 53.1%). Transcriptome for each species was generated using de novo assembly, which was then used as a reference database to evaluate RSV-induced transcriptomic changes in lungs of the infected animals compared to uninfected controls.

### De novo transcriptome assembly of S. fulviventer and S. hispidus reads

For each species, sequenced reads from all 5 tissue types were combined. Trinity^31^ was used for de novo assembly of contigs (also referred to as ‘transcripts’) following in silico normalization to achieve an average coverage of 50x. The assembler generated over 1.3 million contigs per species, which were further filtered based on length^32^ and redundancy^33,34^ (supplementary table 1). The following assembly statistics are shown in Table 1. Each transcript was assigned a unique identifier that designated each ‘gene’ (*S. fulviventer* = 587,619, *S. hispidus* = 559,830) and their alternatively spliced ‘isoforms’ (*S. fulviventer* = 620,569, *S. hispidus* = 592,099). Assembled transcripts from both species had a GC content of 43% and contig N50 > 1600 (which exceeds N50 of other published transcriptome assemblies^35–37^). The Ex90N50, which is the N50 but limited to the top 90% of total normalized transcripts, was 2503 (*S. fulviventer*, #transcripts = 108,995) and 2162 (*S. hispidus*, #transcripts = 240,587) (Table 1).

**Table 1:** De novo assembly and annotations statistics.

Transcriptome assembly	*S. fulviventer*	*S. hispidus*
Contigs/Isoforms	620,569	592,099
Genes	587,619	559,830
Genes (TPM > 1)	270,451	474,882
Median/Mean Contig Length	580/1003.01	597/1032.27
GC%	43.28	43.32
Contig N50	1604	1655
Ex90-N50:#Genes	2503:108,995	2162:240,587
Reads Mapped Back to Assembly	81.9%	87.9%
BUSCO (vertebrata n = 3354)	90.2%	90.4%
**Annotation**		
Identified Coding Sequences	118,159	113,999
Annotated transcripts (*BlastX* 1e-5)	118,060	117,153
Unique genes (*no duplicates*)	18,726	18,380
Annotated proteins (*BlastP* 1e-5)	31,855	30,953
Unique proteins (*no duplicates*)	17,154	16,887
*KEGG* Pathways	90,642	89,654
*Pfam* (protein domains)	5042	6298
*TmHMM* (transmembrane helices)	18,846	18,027
*SignalP* (signaling proteins)	7915	7778
*EggNOG* (orthology relationships)	2902	2205

**Figure 1:**
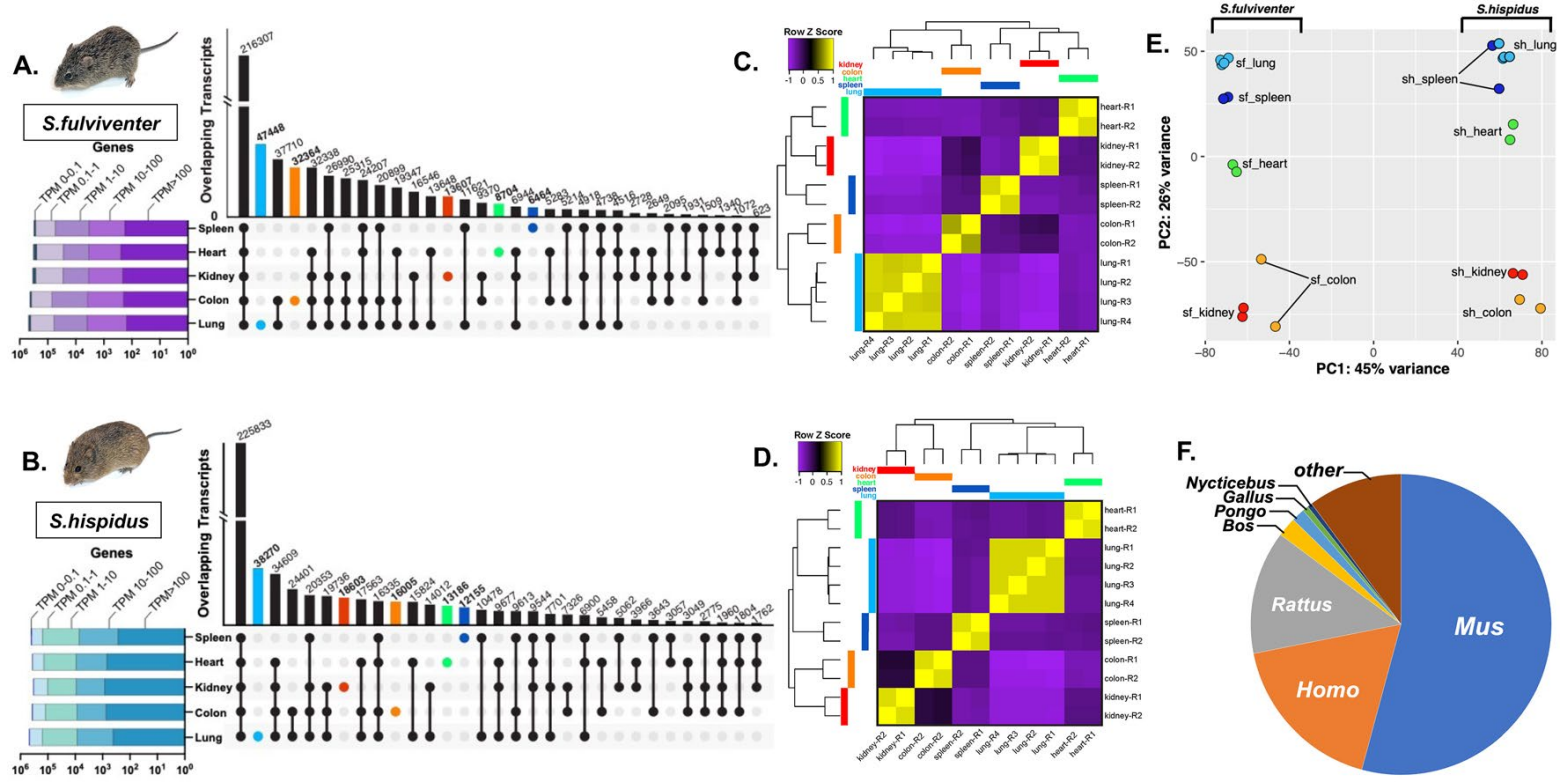
*Sigmodon* transcriptome assembly using Trinity. (**A**,**B**) UpSet plot of overlapping and tissue-specific transcripts expressed in the spleen (blue), heart (green), kidney (red), colon (orange), and lung (blue) of both (**A**) *S. fulviventer* (n = 2) and (**B**) *S. hispidus* (n = 2)*.* Bars for overlapping sequences are shown in black, while bars for sequences unique to individual organs are shown in corresponding colors. Number of overlapping transcripts are shown above each bar, with individual organ comparison on the y-axis. Number of unique genes per tissue are represented by the horizontal barplot. (**C**,**D**) Hierarchical clustering and heatmap by Z-score of all differentially expressed transcripts (Q < 0.001,L2fc > 2, Euclidean distance) within individual tissue samples of both (**C**) *S. fulviventer* and (**D**) *S. hispidus.* (**E**) Principal component analysis (PCA) of expressed transcripts within healthy tissue of both species. (**F**) Taxonomic source of gene annotations determined by NCBI BlastX. Data represents annotations of *S. fulviventer* transcriptome; *S. hispidus* not shown but varies by ~ 1% in all categories except “other”.

The number of transcripts and their expression levels varied between tissue type, and several transcripts were tissue-specific, with the lung having the greatest number of unique transcripts (*S. fulviventer* = 47,448, *S. hispidus* = 38,270) (Fig. 1A,B). Other transcripts were either found in all 5 tissues (*S. fulviventer* = 216,307, *S. hispidus* = 225,833) or a combination of two or more tissue types (Fig. 1A,B). Many transcripts were differentially expressed between tissues (*S. fulviventer* = 49,215, *S. hispidus* = 55,415) as determined by DESeq2 and visualized by hierarchical heatmap clustering^38^ (Fig. 1C,D). The principal component analysis shows significant difference in gene expression patterns between species and different tissue samples, indicated by strong clustering based on species and tissue type (Fig. 1E). Following assembly, transcripts were annotated using protein sequence database (SwissProt) to search for homology against other species. For both species, transcripts shared the highest homology with *Mus musculus* (54%), *Homo sapiens* (17%) and *Rattus rattus* (13%) (Fig. 1F).

To assess the quality of our assembly (Table 1), we mapped the full assembly back to the quality-trimmed reads using Bowtie2^39^ to find 81.9% and 87.9% of reads were utilized during assembly of the *S. fulviventer* and *S. hispidus* transcriptome respectively, for which > 70% is indicative of good quality. We also assessed transcriptome completeness by searching for evolutionarily-conserved BUSCO^40^ groups from the vertebrata_odb10 lineage dataset (total n = 3354) within our dataset. The *S. fulviventer* assembly had 92.7% complete BUSCOs (Complete:3109 [Complete&Single:785, Complete&Duplicated:2324], Fragmented:154, Missing:91), and *S. hispidus* had 90.2% complete BUSCOs (Complete:3025 [Complete&Single:2599, Complete&Duplicated:426], Fragmented:194, Missing:135). All assembly quality statistics are also shown in Table 1. Final reference transcriptome assemblies for each species are made available as supplementary file 1 (*S. hispidus*) and supplementary file 2 (*S. fulviventer*).

### Annotation and functional properties of the S. fulviventer and S. hispidus multi-tissue transcriptome

Transcripts were functionally annotated using the Trinotate pipeline (https://trinotate.github.io/). Transcriptome references were generated for each species independently. First, cotton rat transcripts were aligned to a non-redundant protein annotation database (UniProt^41^)–which contains sequences from 14,132 different taxa (http://web.expasy.org/docs/relnotes/relstat.html)–based on sequence homology via BLASTx^42^ (cutoff e-value < 1e-05). We annotated 118,060 transcripts comprising 18,726 unique genes for *S. fulviventer* and 117,153 transcripts comprising of 18,380 unique genes for *S. hispidus* out of the ~ 600,000 transcripts assembled for each species (Table 1). Coding regions were then identified based on open reading frames (*S. fulviventer* = 118,159, *S. hispidus* = 113,999). Protein coding transcripts were annotated using BLASTp^42^ (cutoff e-value < 1e05) against the UniProt database^41^ to identify > 30,000 annotated proteins per species (~ 17,000 proteins after filtering duplicate annotations). Proteins were further annotated based on protein domains (> 5000), transmembrane helices (> 18,000), and signaling proteins (> 7700). All annotation statistics can be found in Table 1, and the full annotation report can be viewed in supplementary file 3.

**Figure 2:**
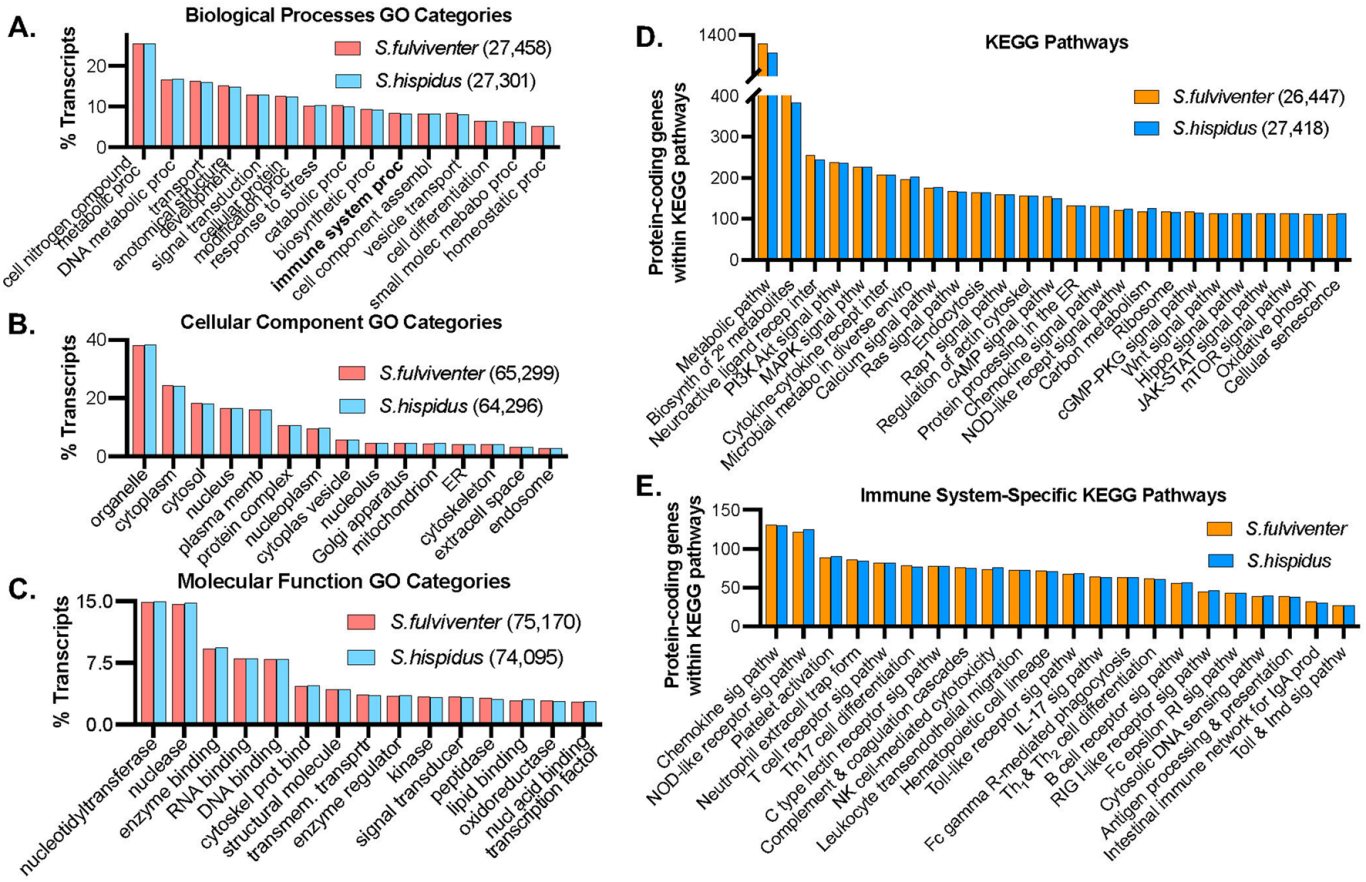
*Sigmodon* transcriptome annotation. (**A**) Biological processes, (**B**) cellular components, and (**C**) molecular function SwissProt Gene Ontology (GO) terms on the x-axis (total GO’s within respective categories including overlaps represented in figure legends) with number of GO’s (percentage of total ~ 107 k annotated transcripts) on the y-axis; GO’s assigned against TrEMBL/SwissProt database using Trinotate v.3.2.2. (**D**) Top 25 KEGG and (**E**) immune system-specific pathways on the x-axis with total number of protein-coding genes on the y-axis; pathways assigned using TransDecoder-determined CDS followed by GhostKOALA (https://www.kegg.jp/ghostkoala/).

In addition to gene name annotations, BLAST alignment to UniProt also assigned several functional terms to describe genes, including Kyoto Encyclopedia of Genes and Genomes (KEGG) Pathways^43^, Gene Ontology (GO)^44^, and EggNOG orthology^45^. Gene Ontology (GO) terms describe the functional properties of genes and their relationship to one another. One or more GO Terms were assigned to 98.35% *S. fulviventer* annotated transcripts (n = 116,115) and 98.45% *S. hispidus* annotated transcripts (n = 115,332). GO terms were grouped into 3 categories: Biological Processes, Cellular Components, and Molecular Function (Fig. 2). Genes from both *S. fulviventer* and *S. hispidus* had a similar ratio of associated GOs. The top Biological Processes GOs included “cellular nitrogen compound metabolic processes” (~ 25%), “DNA metabolic processes” (~ 17%), “transport” (~ 16%), and “anatomical structure development” (~ 15%) (Fig. 2A). Cellular Component GOs consisted mostly of “organelles” (~ 38%), “cytoplasm” (~ 24%), “cytosol” (~ 18%), and “nucleus” (16%) (Fig. 2B). The most common Molecular Function GOs include “nucleotidyltransferase” (~ 15%), “nuclease” (~ 15%), “enzyme binding” (~ 9%), and “nucleic acid binding” (RNA = 8%, DNA = 8%) (Fig. 2C).

**Figure 3:**
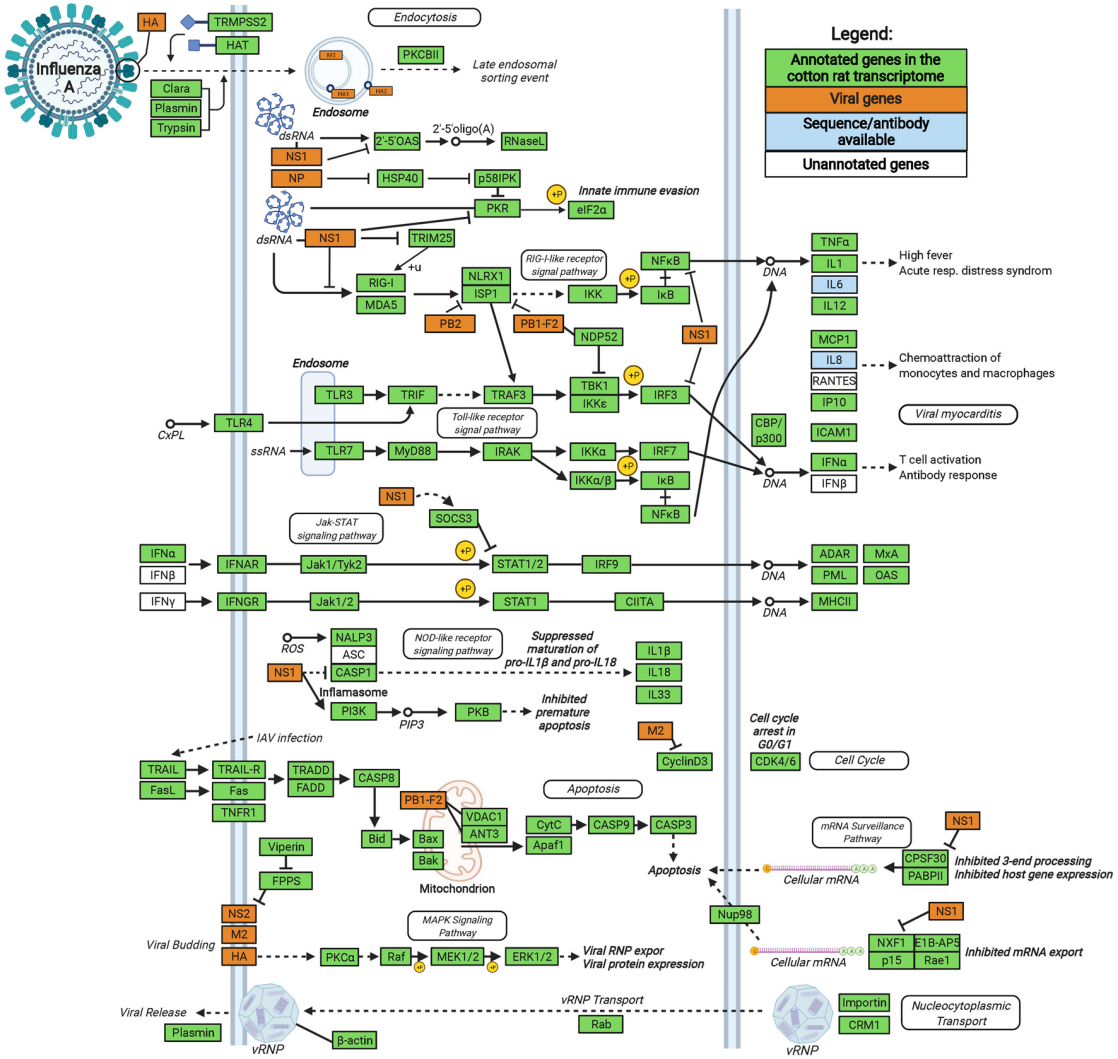
The Influenza Disease Pathway (adapted from Kyoto Encyclopedia of Genes and Genomes [KEGG]). 99 of the 105 essential genes of influenza pathogenesis were successfully assembled and annotated in our de novo transcriptome of *S. fulviventer* and *S. hispidus*. Created with BioRender.

The majority of KEGG pathways identified belonged to metabolism and signaling, specifically secondary metabolite biosynthesis, ligand/cytokine/metal receptor interactions, and a variety of signaling pathways (Fig. 2D, also see Figure S1A for distribution of “signaling and membrane transport” KEGG pathways). We examined immune-specific KEGG pathways to showcase the large number of relevant genes identified in the de novo transcriptome, including chemokine signaling, innate and adaptive cell receptor signaling, complement cascades, and lymphocyte differentiation (Fig. 2E). KEGG also provides gene mapping against many infection pathways, including infectious agents successfully modeled in cotton rats i.e., influenza^18^, HIV-1^29^, and measles^22^ (Figure S1B). We mapped gene annotations of both *S. fulviventer* and *S. hispidus* to the host genes associated with influenza infection KEGG pathway, which encompassed 94.3% of the genes required during influenza infection (Fig. 3). All other pathways can be reconstructed and visualized using KEGG’s reconstruct tool (https://www.kegg.jp/kegg/mapper/reconstruct.html) using data included in the supplementary file 4.

### Histopathology and gene expression changes after RSV infection in the lung of S. fulviventer and S. hispidus

**Figure 4:**
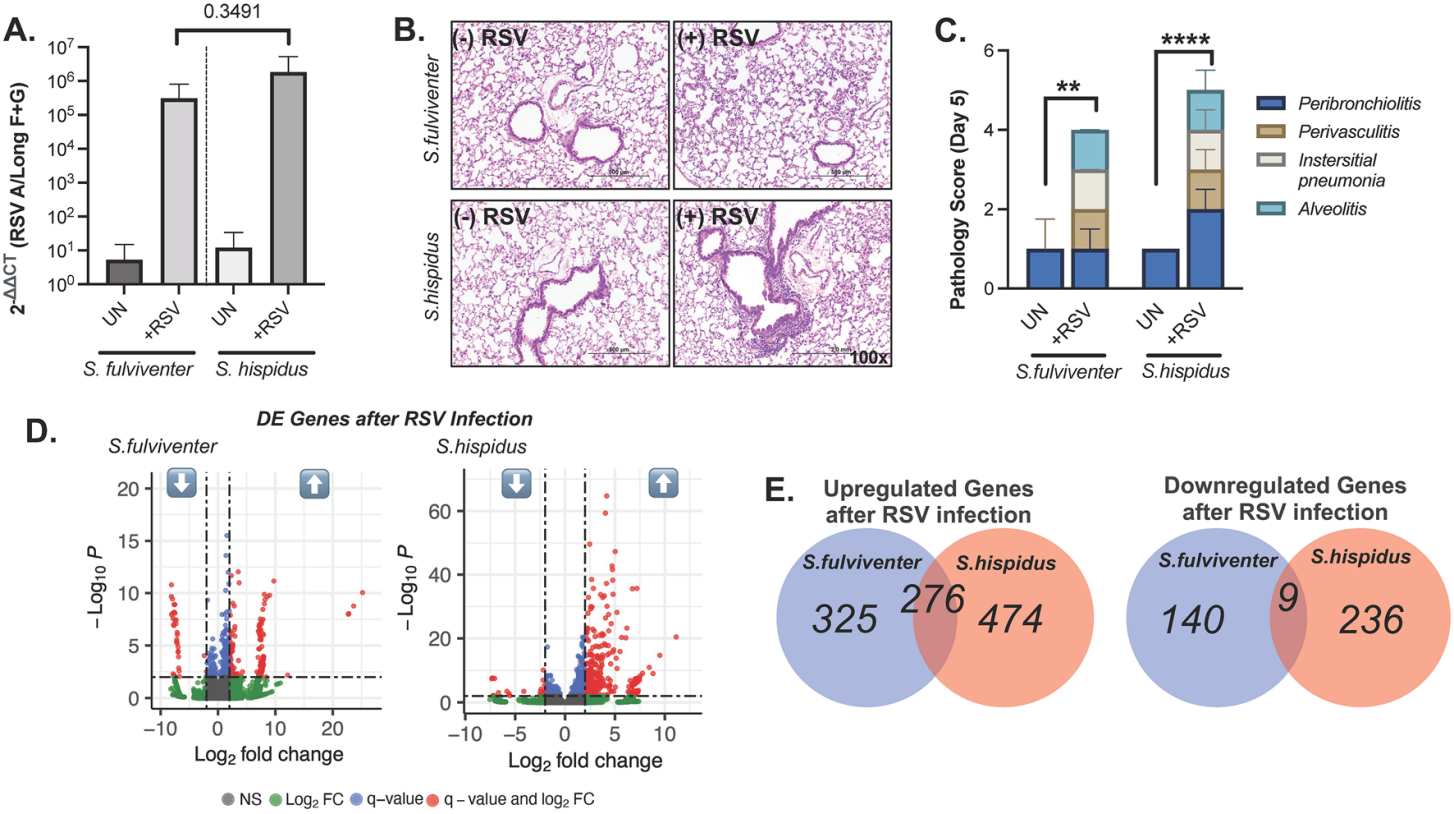
RSV-induced changes to the lung environment. (**A**) Viral titers (RSV G and F proteins) in uninfected and infected lungs determined by qRT-PCR normalized to β-actin; fold change (calculated by 2^−ΔΔCT^) on the y-axis. (**B**) Histopathological imaging and (**C**) blindly generated pathology scoring for both healthy (n = 4/species) and RSV-infected (n = 5/species) lung tissue from *S. fulviventer* and *S. hispidus*. (**D**,**E**) DESeq2 analysis of assembled genes from lung tissues (same tissue imaged from Figures A-B) revealed several genes that are differentially expressed (DE) between healthy and infected tissues (*p* < 0.05, q < 0.05, l2fc >|1.0|) of *S. fulviventer* (blue) and *S. hispidus* (red), of which several genes were successfully annotated using Trinotate (as indicated in boxes). *p ≤ 0.05, **p ≤ 0.01, ***p ≤ 0.001, ****p ≤ 0.0001.

We intranasally infected *S. fulviventer* and *S. hispidus* with 10^5^ PFU of RSV A/Long or PBS (as mock infection control) and harvested lungs after 5 days post infection for analysis. Infection was confirmed by qRT-PCR targeting the RSV G and F mRNA, where significantly higher RNA copy was found in infected animal, whereas uninfected controls did not have amplification of viral RNAs (Fig. 4A). There was no significant difference in viral RNA levels between *S. fulviventer* and *S. hispidus* following 5-day infection (*p* = 0.3491, Tukey’s multiple comparisons test). Lung tissue was evaluated for histopathology (Fig. 4B) and blindly scored for 4 inflammatory parameters as described in the methods: peribronchiolitis, perivasculitis, interstitial pneumonia, and alveolitis (Fig. 4C). Each infected animal had a significantly larger cumulative pathology score compared to uninfected controls, indicating successful infection (*Sf* *p* = 0.0020, *Sh* *p* =  < 0.0001, Tukey’s multiple comparisons test) (Fig. 4C). RNA was then sequenced as described, and reads were aligned to the previously annotated reference transcriptome for each species. Following expression normalization, gene expression analysis showed RSV infection significantly altered expression patterns. RSV infection in the lungs of *S. hispidus* resulted in greater alterations in gene expression than that of *S. fulviventer* (Supplemental Figure 2).

Gene differential expression analyses (RSV A/Long vs. mock-infected Controls) identified several genes that were differentially up- and down-regulated in infected lungs, including genes unique and common to each cotton rat species (Fig. 4D). RSV infection in *S. fulviventer* resulted in 325 unique upregulated genes (98 annotated) and 140 unique downregulated genes (20 annotated). RSV infection in *S. hispidus* resulted in 750 upregulated genes (44 annotated) and 245 downregulated genes (8 annotated) (Fig. 4D,E, full data in supplementary file 5). By combining infection data from both species, we found that 276 genes (12 annotated) were upregulated in both species while 9 genes (0 annotated) were downregulated in both species. Only annotated genes for each species were selected for further analysis. Twenty of the top-most differentially expressed annotated genes (selected based on function and Gene Ontology) are listed in Table 2 (*S. fulviventer*) and Table 3 (*S. hispidus*).

**Table 2:** Twenty notable differentially expressed genes in *S. fulviventer* lung following RSV infection.

Gene	Description	Transcript ID	Log2FC	*p* value	q value
*HVM44*	Ig heavy chain V region PJ14	*Sfulv_DN86481_c0_g1*	↓ − 3.51	1.7E−04	3.4E−02
*CREB1*	Cyclic AMP-responsive element-binding protein 1	*Sfulv_DN150233_c0_g1*	↓ − 1.94	1.5E−05	6.3E−03
*CRY2*	Cryptochrome-2	*Sfulv_DN8103_c29_g1*	↓ − 1.69	4.8E−08	5.8E−05
*K2C8*	Keratin, type II cytoskeletal 8	*Sfulv_DN18044_c9_g1*	↓ − 1.54	5.4E−07	4.7E−04
*OCLN*	Occludin	*Sfulv_DN14349_c3_g1*	↓ − 1.49	2.7E−05	9.6E−03
*CPNE3*	Copine-3	*Sfulv_DN28516_c1_g1*	↓ − 1.27	1.6E−04	3.4E−02
*MOV10*	Putative helicase MOV-10	*Sfulv_DN357483_c0_g1*	↓ − 1.14	1.8E−05	7.1E−03
*MFGM*	Lactadherin	*Sfulv_DN100738_c3_g1*	↓ − 1.07	3.4E−05	1.1E−02
*ISG15*	Interferon-induced ubiquitin-like protein, 15 kDa	*Sfulv_DN2597_c2_g1*	↑ 1.02	1.3E−04	2.9E−02
*IIGP1*	Interferon-inducible GTPase 1	*Sfulv_DN1249_c0_g1*	↑ 1.33	3.0E−05	1.0E−02
*OAS1A*	2′-5′-oligoadenylate synthase 1A	*Sfulv_DN24798_c0_g1*	↑ 1.58	7.8E−05	2.0E−02
*I27L2*	Interferon alpha-inducible protein 27-like protein 2	*Sfulv_DN13614_c0_g1*	↑ 1.96	8.0E−13	5.2E−09
*COX1*	Cytochrome c oxidase assembly protein COX15 homolog	*Sfulv_DN104408_c3_g1*	↑ 2.04	4.6E−08	5.7E−05
*CXCL10*	C-X-C motif chemokine 10	*Sfulv_DN18689_c0_g1*	↑ 2.60	6.1E−09	1.0E−05
*PGDH*	15-hydroxyprostaglandin dehydrogenase [NAD( +)]	*Sfulv_DN56601_c1_g1*	↑ 2.95	5.8E−10	1.3E−06
*MX2*	Interferon-induced GTP-binding protein Mx2	*Sfulv_DN17914_c1_g1*	↑ 3.48	1.1E−05	4.8E−03
*EXOC5*	Exocyst complex component 5	*Sfulv_DN7267_c6_g1*	↑ 4.02	4.1E−05	1.3E−02
*UN93B*	Protein unc-93 homolog B1	*Sfulv_DN114523_c0_g1*	↑ 4.04	3.2E−05	1.1E−02
*CFAB*	Complement factor B	*Sfulv_DN212381_c0_g2*	↑ 6.99	1.3E−07	1.4E−04
*GLYC*	Major surface glycoprotein G_*Orthopneumovirus*	*Sfulv_DN7526_c1_g1*	↑ 9.74	2.5E−16	7.1E−12

Negative Log2FC = downregulated, positive Log2FC = upregulated. Gene names/descriptions determined by BlastX against UniRef/SwissProt. Transcript IDs correspond to contig names (Sfulv_xxx) in assembly FASTA file. Log2FC = log2FoldChange, q = FalseDiscoveryRate.

**Table 3:** Twenty notable differentially expressed genes in *S. hispidus* lung following RSV infection.

Gene	Description	Transcript ID	Log2FC	*p* value	q value
*LMTD1*	Lamin tail domain-containing protein 1	*Shisp_DN10656_c2_g1*	↓ − 1.04	3.7E−04	3.5E−02
*NLRC5*	NLR family CARD domain containing 5	*Shisp_DN1039_c0_g1*	↑ 1.74	1.5E−06	3.3E−04
*GBP4*	Guanylate binding protein 4	*Shisp_DN1171_c0_g1*	↑ 1.88	2.8E−13	2.3E−10
*IFIT1*	Interferon induced protein with tetratricopeptide repeats 1	*Shisp_DN1078_c15_g1*	↑ 2.01	2.6E−06	5.3E−04
*LY6E*	Lymphocyte antigen 6 family member E	*Shisp_DN101026_c0_g1*	↑ 2.20	2.0E−12	1.4E−09
*IRF9*	Interferon regulatory factor 9	*Shisp_DN12125_c1_g1*	↑ 2.33	3.2E−07	8.4E−05
*C4BPA*	C4b-binding protein alpha chain	*Shisp_DN107540_c0_g1*	↑ 2.42	5.6E−13	4.3E−10
*CO4A*	Collagen alpha-1(IV) chain	*Shisp_DN122397_c0_g1*	↑ 2.43	3.1E−05	4.6E−03
*LG3BP*	Galectin-3-binding protein	*Shisp_DN11955_c0_g1*	↑ 2.45	1.1E−12	8.1E−10
*PSB9*	Proteasome subunit beta type-9	*Shisp_DN13011_c0_g1*	↑ 2.52	5.2E−10	2.3E−07
*K2C6A*	Keratin, type II cytoskeletal 6A	*Shisp_DN12075_c0_g1*	↑ 2.70	1.5E−05	2.4E−03
*HSH2D*	Hematopoietic SH2 domain-containing protein	*Shisp_DN0_c15_g8*	↑ 2.80	3.0E−12	2.1E−09
*ABEC1*	C- > U-editing enzyme APOBEC-1	*Shisp_DN11754_c2_g1*	↑ 3.45	3.9E−25	1.0E−21
*RSAD2*	Radical S-adenosyl methionine domain-containing prot. 2	*Shisp_DN112437_c0_g1*	↑ 3.51	3.5E−12	2.3E−09
*CXCL11*	C-X-C motif chemokine 11	*Shisp_DN149434_c0_g1*	↑ 3.56	8.3E−15	8.1E−12
*MX2*	Interferon-induced GTP-binding protein Mx2	*Shisp_DN140604_c0_g1*	↑ 4.24	1.0E−18	1.5E−15
*CXCL10*	C-X-C motif chemokine 10	*Shisp_DN14446_c0_g1*	↑ 4.62	6.3E−29	2.5E−25
*OAS3*	2′-5′-oligoadenylate synthase 3	*Shisp_DN1184_c1_g1*	↑ 5.12	1.3E−17	1.6E−14
*I27L2*	Interferon alpha-inducible protein 27-like protein 2	*Shisp_DN12103_c7_g1*	↑ 6.20	1.6E−27	6.0E−24
*SYWC*	Tryptophan–tRNA ligase, cytoplasmic	*Shisp_DN144721_c0_g1*	↑ 8.44	1.1E−14	1.1E−11

Negative Log2FC = downregulated, positive Log2FC = upregulated. Gene names/descriptions determined by BlastX against UniRef/SwissProt. Transcript IDs correspond to contig names (Shisp_xxx) in assembly FASTA file. Log2FC = log2FoldChange, q = FalseDiscoveryRate.

### Species-specific changes to the RSV-infected lung

In *S. fulviventer*, several genes were downregulated upon infection related to immunoglobulin structure (*HVM44*), transcription factors (*CREB1*, *CRY1*), epithelial structure integrity (*K2C8*), formation and maintenance of tight junctions (*OCLD*), membrane trafficking regulation (*CPNE3*), targeted RNA degradation (*MOV10*), and tissue remodeling and homeostasis (*MFGM*). *GLYC*, which is the RSV G protein, was marked as upregulated in RSV lungs due to the absence of reads in the uninfected group (indicated by DESeq2 basemean = 0). Upregulated host genes were related to the complement system (*CFAB*), immune receptor signaling (*UN93B*), vesicle trafficking (*EXOC5*), interferon-induced antiviral activity (*MX2, CXCL10, I27L2, OAS1A, IIGP1, ISG15*), prostaglandin metabolism (*PGDH*), and electron transport (*COX1*). These differentially expressed *S. fulviventer* genes following RSV infection are listed in Table 2.

In *S. hispidus*, only 1 annotated gene was downregulated: *LMTD1*, which is involved in cell proliferation. Other downregulated genes had annotations indicating inclusion of signaling peptides (via SignalP) and transmembrane regions (via TmHMM) but with unknown function (Supplementary File 5). Several upregulated immune genes in *S. hispidus* were also upregulated in *S. fulviventer* (*I27L2, CXCL10, MX2*). Most upregulated genes were related to cytokine stimulation of antiviral activity (*OAS3, CXCL11, IRF9, IFIT1, NLRC5*), proteasomal degradation (*PSB9*), cytoskeletal reorganization in response to stress (*SYWC*, *K2C6A*), T cell activation (*HSH2D, LY6E*), post-transcriptional regulation (*ABEC1*), viral protein degradation (*RSAD2*/*Viperin*), complement pathway (*CO4A, C4BPA*), chemical metabolism (*GBP4*), and cellular defense-related signal transduction (*LG3BP*). These differentially expressed *S. hispidus* genes following RSV infection are presented in Table 3.

Of the differentially expressed genes (DEGs) following RSV infection, 12 DEGs were found to be either up- or down-regulated in both *S. fulviventer* and *S. hispidus*. The chemokine *CXCL10*, *MX2*, and *I27L2* were among the top 10 upregulated genes in both species. Other DEGs within our significance threshold include *GBP2, GBP4, IRF9, NLRC5, OAS3, PAR14, RN213, SYWC,* and *IIGP1.* All DEs can be found in Supplementary File 5.

**Figure 5:**
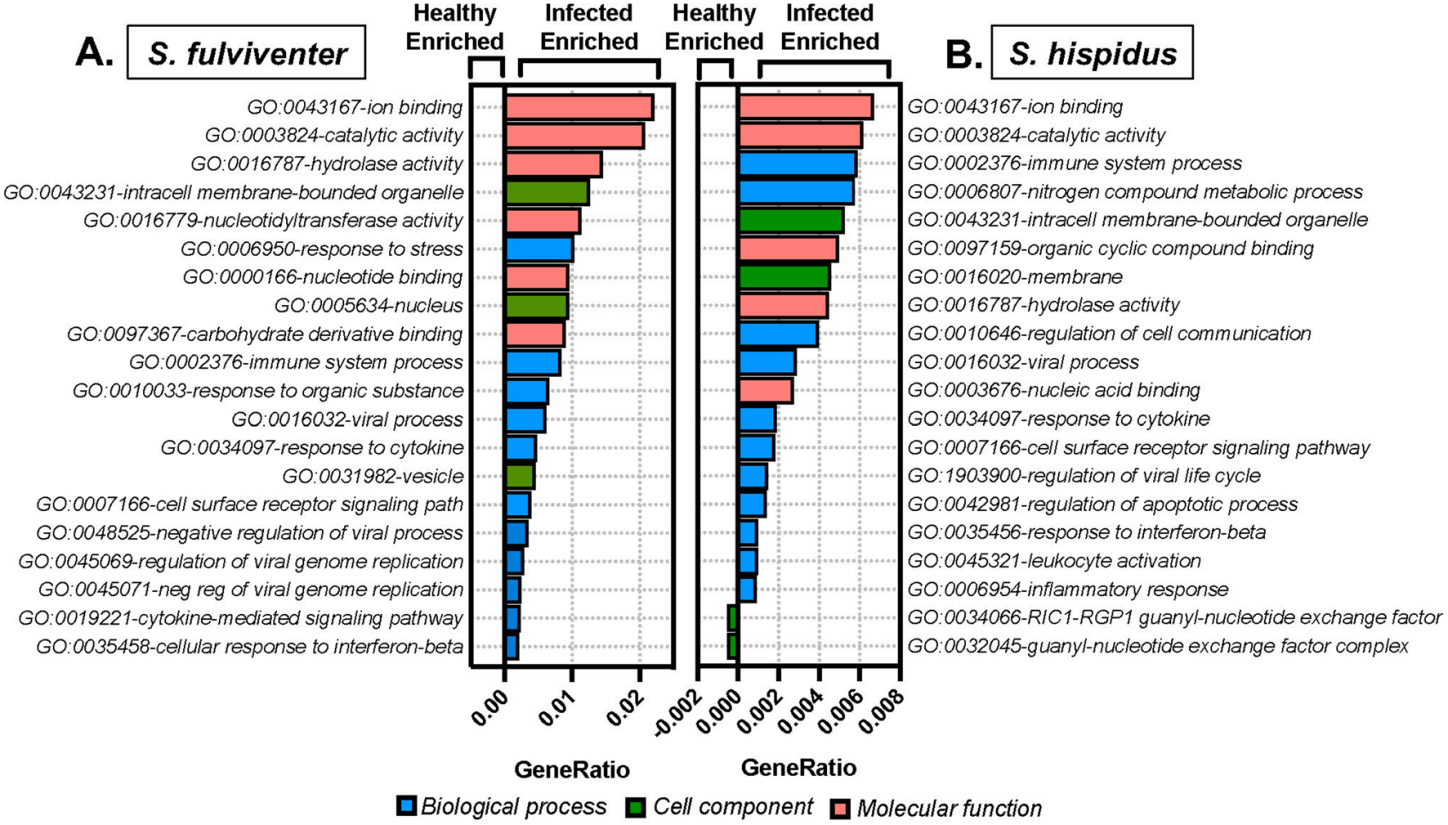
Categorical changes in gene expression after RSV infection in both (**A**) *S. fulviventer* and (**B**) *S. hispidus* using Gene Ontology (GO) annotations and GoSeq. SwissProt GO terms on the y-axis (color coordinated based on biological process [blue], cell component [green], or molecular function [red]) with relative expression of GOs on the x-axis (expressed as GeneRatio, which is the proportion of differentially expressed genes in each GO category to the total number of differentially expressed genes in all significant GOs). Positive GeneRatio = enriched in RSV-infected lungs; negative GeneRatio = enriched in healthy lungs (no significant GOs in healthy *S. fulviventer*). All GOs were statistically significant (*p* < 0.05, q < 0.05).

Additionally, differentially expressed Gene Ontology (GO) terms were determined using the GoSeq R package^46^. The top GOs enriched in infected lungs of both *S. fulviventer* (Fig. 5A) and *S. hispidus* (Fig. 5B) were “biological processes” such as response to cytokine and interferon-beta, immune system process, cell surface receptor signaling pathway, and viral process; “molecular functions” such as ion binding, catalytic activity, hydrolase activity, and “cellular components” such as intracellular membrane-bounded organelles and nucleus. Other enriched GOs in infected lungs were related to enhancing the components and function of cells, such as components of the membrane and vesicle, response to stress, regulation of apoptotic processes, regulation of viral genome replication, leukocyte activation, and binding of carbohydrates and other organic compounds. Only *S. hispidus* had enriched GOs in healthy tissues, and these included the cellular component involved in the guanyl-nucleotide exchange factor complex.

### Confirmation of differentially expressed genes

To confirm DEGs three de novo assembled genes were selected based on their upregulation on day 5 post infection (*Shisp_DN132151_c0_g1* [*IIGP1*], *Shisp_DN12103_c7_g1* [*I27L2*], *Sfulv_DN158_c1_g1* [*IIGP1*]). qRT-PCR with SYBR green was performed with primers designed to target each gene, normalized to the β-actin housekeeping gene. The three genes included IIGP1 in both *S. fulviventer* and *S. hispidus* and *I27L2* in *S. hispidus*. qRT-PCR confirmed gene upregulation post RSV infection (i.e., positive fold changes) that correlated with the RNA-Seq data (Supplementary Table 2).

Additionally, to further confirm and validate published differential expression analysis by Rajagopala et al.^27^, where *S. hispidus* was infected with RSV A/Long in the same facility with the same strain and concentration of virus, we compared with DEGs identified in this study albeit samples were collected in different days. Rajagopala et al.^27^ study included two time points; Day 4 and 6 post-infection, whereas, in this study we have collected sample only on Day 5. However, the DESeq2 analysis parameters used in both studies are same. Compared to the Rajagopala et al.^27^, this current study confirmed 4 of the 5 Eukaryotic DEGs (*IFIT1, MX2, OASL2, RSAD2*) that were modified on day 4. Similarly, this study confirmed 29 of the 81 DEGs following infection by RSV (including *CFAB, GBP2/4/5/6, IFIT1, IIGP1, K2C6A, MX2, OASL2, RSAD2*; full data in Supplementary File 5) that were modified on day 6.

## Discussion

Cotton rats are excellent pre-clinical animal models for the study of infections with RSV and other viruses, but without a published genome for either species, genetic or transcriptomic analysis in cotton rats is limited. In this study, we generated comprehensive transcriptomes from multiple organs of two inbred species of cotton rats: *S. fulviventer* and *S. hispidus*. Comparison of our two transcriptomic references revealed large differences in transcript sequence homology and baseline gene expression levels between each species, although the total number of annotated genes and functional categories (gene ontology, KEGG terms) were similar (Table 1).

Our transcriptome assembly and annotation surpass the quality and completeness standards of previously published transcriptome references in other animals^35,36,47^. The quality and depth of our transcriptome assembly also improved upon our previous lung transcriptome of *S. hispidus* lung tissues^27^ due to inclusion of multiple tissues. This resulted in nearly 3 times the number of annotated transcripts (117,153 vs. 38,736) and an additional 6169 unique gene annotations in *S. hispidus*. Furthermore, our application of the transcriptome reference to infer RSV-induced alterations of gene expression exhibits the utility of our reference in the study and treatment of many infections, including RSV, influenza, and other respiratory viruses.

Our analysis confirmed many genes previously implicated in RSV infection and immunity. *RSAD2* (or Viperin) is a significantly upregulated gene upon RSV infection in *S. hispidus* that inhibits RSV filament formation and cell–cell viral transmission without inhibiting viral protein expression^48^. Viperin is also the most upregulated gene in human nasal epithelium following intranasal challenge with Rhinovirus^49^. Another RSV-related gene is *I27L2*, the top upregulated gene in *S. hispidus* and *S. fulviventer* that is also the top differentially expressed gene in preterm infants with severe RSV infection^50^. Other upregulated genes also shown to be involved in RSV infection include chemokines *CXCL10*^51^, *CXCL11*^52^, *LY6E*^53^, *MX2*^54^, *OAS1A*^55^, *ISG15*^56^*, IRF9*^57^*, NLRC5*^58^ and K2C6A^59^. We also identified genes involved in the complement system (*CFAB, CO4A, C4BPA*) and prostaglandin metabolism (*PGDH*), both of which are involved in the host response to RSV^60,61^. The results from our study supports the importance of these genes in host-mediated protection against RSV and the cotton rat’s translational value in viral research.

*CRY2* (downregulated in *S. fulviventer*) has also been indirectly attributed to RSV disease severity. *CRY2* is a transcription factor that modulates circadian rhythms through suppression of *BMAL1*, a putative regulator of cellular factors essential for viral replication^62^. Analysis of *BMAL1*-/- primary cells revealed low-*BMAL1* expression enhances susceptibility to influenza^63^, parainfluenza virus 3, and RSV^64^. *BMAL1* also shows seasonal variation in humans with lowest levels in winter months during peak respiratory virus season^62^. While this gene will need further investigation in its role in RSV pathogenesis, this is the first association of modulated expression during RSV infection.

The cotton rat mimics the respiratory infections in humans due to the presence of human homologous genes that are absent in other laboratory rodents (e.g. *Mus musculus*), i.e., interferon-stimulated *MX* genes^65^. Our analysis is consistent with the upregulation of *MX2* in both *S. fulviventer* and *S. hispidus* (as well as *MX1*, *MX1B*, and *MX3* in *S. fulviventer*) upon RSV infection as previously described^66^, highlighting the importance of this model in capturing interferon-induced immune response to RSV and other viruses. Additionally, our analysis captures *IIGP1* upregulation in *S. fulviventer* and *S. hispidus*. *IIGP1* is another human interferon-stimulated gene with similar importance and function of *Mx* genes, but it is only present in mice as a less-effective paralog *IGPT*^67^. A study on IGPT-deficient mice found no increased susceptibility or interferon-induced cytokine production towards intracellular pathogens such as *Listeria* and cytomegalovirus, which suggests a different upstream mechanism in mice^68^. *We, for the first time, successfully annotated IIGP1 in cotton rats and show its modulation during RSV infection. While functional validation studies are needed, results from our study further argue the translational utility of the cotton rat as a model for RSV and other respiratory viruses compared to mice, who are deficient in IIGP-1*.

*CREB1* (cAMP-responsive element-binding protein 1) is a widely studied transcriptional factor that modulates many immune genes and was downregulated in *S. fulviventer* upon RSV infection*. CREB1* promotes anti-inflammatory responses such as inhibition of NF-κB activity, the induction of IL-10, and the generation of Tregs^69^. *CREB1* activation was observed to be a driver of vaccine efficacy in HIV-1 vaccines in nonhuman primates by recruiting CD4 + T cells and B cells to the site of antigen presentation^70^. Future functional studies are needed to further understand whether there is an association between RSV and CREB1.

One more interesting upregulated gene in both *S. fulviventer* and *S. hispidus*, *SYWC* is an interferon-induced activator of ERK, Akt, and eNOS pathways and cytoskeletal reorganization in response to stress and is a serum marker for pulmonary tuberculosis^71^. While this study is the first association of *SYWC* with RSV infection, further study may uncover it as a marker for severe RSV infection in tissue and serum.

While we have presented a comprehensive overview of the cotton rat transcriptome, we recognize some limitations in our study. Previously published data from Pletneva et al. has shown that different RSV strains and isolates have differential induction of interferon activated genes in cotton rats^66^*.* While the study by Pletneva et al. only includes *S. hispidus*, we expect that this is also true for *S. fulviventer* due to similar susceptibility to RSV A/Long. To this end, we recognize that our conclusions are limited to RSV A/Long, and transcriptomic comparison of RSV variants should be considered for future studies. Further, we identified multiple genes that have not been previously implicated in RSV infection, such as (*LG3BP, SYWC, ABEC1, IIGP1, CREB1*). While our analysis suggests potential unknown mechanisms of pathogenesis, we recognize that association of these genes with RSV infection is severely limited in the absence of experimental data. Such efforts are beyond the scope of this paper on the reference cotton rat transcriptome but will be considered for future studies.

## Conclusion

This report highlights the utility of cotton rat transcriptome to understand infection-induced transcriptomic changes in the absence of an available genome references. Both *S. hispidus* and *S. fulviventer* transcriptome references have been made publicly available at BioProject PRJNA816878 for additional research. Additionally, identification of gene sequences allows for better understanding of cotton rat genetics and generation of molecular tools, such as qRT-PCR primers and probes targeting genes of interest, recombinant protein, antibodies, and other assays. Differential gene expression analysis revealed host species specific differences upon RSV infection. As the development of RSV therapeutics calls for a well-developed, robust pre-clinical models for RSV, we hope our transcriptome references and gene associations with RSV can accelerate biomedical interventions against this pathogen of significant public health importance.

## Methods

### Animals, infection, and tissue collection

Four- to six-week-old *S. hispidus* (n = 8 male, 1 female) and *S. fulviventer* (n = 8 male, 1 female) cotton rats (~ 100 g) were obtained from the inbred colony maintained at Sigmovir Biosystems, Inc. Cotton rats in the colony were seronegative by ELISA to adventitious respiratory viruses (*i.e.,* Pneumonia Virus of Mice, Rat parvovirus, Rat coronavirus, Sendai virus). Each species was randomly split into 2 groups: RSV-infected (n = 5) and Uninfected controls (n = 4). Transcriptome assembly and annotation was performed on all tissues (whole lung, large intestine, heart, spleen, and kidney) from 2 males within each uninfected group. Female animals were only included in RSV infected groups. To avoid fighting, all animals were individually housed in large polycarbonate cages and fed a diet of standard rodent chow and water ad libitum. All animal procedures followed NIH and USDA guidelines approved by the Sigmovir Biosystems, Inc. IACUC. Study design, analysis, and reporting of methods and results are presented in accordance with the ARRIVE guidelines.

The Long strain of RSV A/Long (ATCC Cat. # VR-26) was propagated in HEp-2 cells, and viral titer was determined using plaque assay. Cotton rats were intranasally inoculated under isoflurane anesthesia with 10^5^ plaque forming units in 100 µL of either RSV suspension or PBS vehicle. Animals were sacrificed by carbon dioxide inhalation at day 5 of infection. Whole lung, large intestine, heart, spleen, and kidney were dissected from all animals and frozen in RNA-later (Invitrogen AM7021) for processing and sequencing at VUMC.

### Lung histopathology and viral titers

Lungs (right lobe) were dissected and inflated with 10% neutral buffered formalin and immersed in formalin for fixation. Lungs were embedded in paraffin blocks, sectioned, and stained with hematoxylin and eosin (H&E). Pathologists were blinded to the study group, and slides were examined for 4 parameters of pulmonary immune cell infiltration and inflammation as previously described^72^: peribronchiolitis (bronchioles), perivasculitis (small blood vessels), interstitial pneumonia (alveolar walls), and alveolitis (alveolar space) (listed from least to greatest severity). Each condition was assigned a score from 0 to 4, where 0 is no pathology and 4 is max pathology. Scores correspond to the percentage of pathology present in the field of view pictured in Fig. 4A (0 = 0%, 1 = 5%, 2 = 25%, 3 = 75%, and 4 = 100%). Cumulative pathology scores were calculated by summing of the median score for each condition.

Viral RNA copy numbers were determined using Real-Time Quantitative Reverse Transcription Polymerase Chain Reaction (qRT-PCR) from RNA extracted from lung (lingular lobe) homogenates (see next section *RNA extraction and cDNA library preparation* for extraction methods). Following extraction, cDNA was generated using 1ug of total RNA and the SuperScript™ III Reverse Transcriptase (Invitrogen™) kit according to the manufacturer’s instructions. cDNA was diluted 1:5, and 3uL was added to qPCR reactions using iQ™ SYBR® Green Supermix (10uL total). Reactions for each target were performed in duplicate for each sample using primers (IDT, 250 nm final concentration) targeting the G and F mRNA of RSV and β-actin, which have been previously published and validated for use in both species of cotton rats^73^. No-template-controls and a positive control (RNA extracted from RSV A/Long viral stock used for infection) were run on each plate. CT values for both G and F were averaged and normalized to β-actin. Results were calculated using the 2^−ΔΔCT^ method^74^. Figures and statistical analysis were performed using ANOVA/Tukey’s multiple comparisons test in Prism 8.

### RNA extraction and cDNA library preparation

RNA was prepared as previously described^27^. In summary, small sections of cotton rat lung (lingular lobe), large intestines (~ 20 mm colon, flushed with sterile PBS), spleen, kidney, and heart were homogenized using a NextAdvance Bullet Blender® with RED RINO™ tubes containing 3.2 mm stainless steel beads (SSB32) and 2 × volume of QIAzol® Lysis Regent (Qiagen, cat. no. 79306) at max speed for 3 min. RNA was extracted from homogenates using RNeasy® Plus Universal Mini kit (Qiagen, cat. No. 73404) via additional QIAzol® (total volume 900uL), gDNA eliminator, and chloroform with column DNase digestion as recommend by the manufacturer’s protocol. RNA quality was measured using an Agilent 2100 Bioanalyzer (Agilent Technologies, CA, USA). Host rRNA was removed using the NEBNext rRNA Depletion Kit v2 (Cat no: NEB E7400X). The cDNA libraries were prepared using 1 μg total RNA from each sample using the NEBNext Ultra II RNA Library Prep Kit for Illumina (Cat no: NEB E7805L) following the manufacturers protocol for intact RNA (RIN > 7), AMPure XP Beads for cleanup steps (Beckman, cat. No. A63881), and NEBNext Multiplex Oligos for Illumina (Set 1, cat no: E7600S) for pooling samples. Sequencing was performed using Illumina NovaSeq6000 2 × 150 bp sequencing at the Vanderbilt Technologies for Applied Genomics (VANTAGE) core facility.

### Transcriptome assembly

Data was then parsed into individual samples by barcode. Adaptor sequences, low quality (minimum Phred 33), and short (< 75 bp) reads were removed using Trimmomatic (version 0.36, “ILLUMINACLIP: NEB_multiplexoligos.fa:2:30:10 TRAILING:3 SLIDINGWINDOW:4:15 MINLEN:75”)^75^. Only paired-end reads which passed the quality threshold as described in^76^ were retained.

About 921 million paired-end reads from healthy tissues were utilized for de novo transcriptome assembly using the Trinity v2.13.1 software package^31^, with default parameters of 50 × coverage. An additional 314.5 million paired-end reads from viral-infected lungs were used for experimental analysis. Contigs were assembled for *S. fulviventer* and *S. hispidus* by first clustering individual contigs/transcripts into ‘genes’ followed by construction of de Bruijn graphs to report full length, alternatively spliced isoforms. All transcripts were then filtered by transcript length cutoff of < 200 bp using SeqKit^32^, contaminant annotation (Blast annotation of “virus”, “bacteria”, or “fungi”), sequence similarity of 95% or greater using CD-HIT^33^, and removal of redundant mRNAs picked by the EvidentialGene tr2aacds pipeline^34^. Assembly statistics of final transcripts, such as mean number of transcripts, transcript length, mean transcript length, N50, are listed in Table 1. Raw reads and assembly data will be deposited in SRA under BioProject PRJNA816878 upon acceptance of the manuscript for publication.

The RSEM package was used for quantifying and normalizing gene and isoform abundances from paired-end RNA-Seq data^77^. RSEM enables accurate transcript quantification per sample and sample type without a reference genome. TPM (transcript per million mapped reads) was calculated using the RSEM package with Bowtie2 aligner, and a cutoff of TPM > 1 was used to filter the low-quality assembled transcripts to use for differential expression analysis. The Ex90N50 was calculated using the Trinity *contig_ExN50_statistic.pl* script.

### Transcriptome characterization

The Trinotate v3.2.2 annotation pipeline was used to annotate transcripts from both *S. hispidus* and *S. fulviventer*. BlastX and BlastP (e-value cutoff of 1e-05)^42^ were used to find sequence homology of individual contigs and protein coding regions (determined by Transdecoder, https://github.com/TransDecoder/TransDecoder) against the UniProt/SwissProt database^41^ (data found in supplementary file 6). KEGG terms^43^, Gene Ontology terms^44^, and EggNOG terms^45^ from the Swissprot^41^ database alignment with BLAST^42^. Other tools within the pipeline annotated transcripts based on protein domains via Pfam^78^, transmembrane helices via TmHMM^79^, and signaling proteins^80^.

### Differential gene expression and GO terms

Lungs isolated from RSV-infected and Uninfected groups were processed and sequenced as described above. The Trinotate pipeline was used to annotate all reads from experimental groups against our reference. Normalized expression levels of transcripts across tissues were determined using Salmon^81^ and the TPM (transcripts per million) metric; only transcripts with a TPM > 1 were used for downstream differential expression analysis (*S. fulviventer* = 270,451, *S. hispidus* = 474,882). Raw count matrices at the gene level for each species can be found in supplementary file 7 DESeq2 package^38^ was used within the pipeline to by compare experimental group (*RSV-infected lungs*) containing biological replicates with the corresponding control group (*uninfected lungs*). Genes with a *p* < 0.05, adjusted *p* < 0.05 (“q”/false discovery rate/Benjamini-Hochberg), and a log_2_ fold change >|1| were treated as differentially expressed. We used the goseq package in Bioconductor to detect differentially abundant GO terms^44^. GOs with a *p* < 0.05 and adjusted *p* < 0.05 (“q”/false discovery rate/Benjamini-Hochberg) were treated as differentially expressed.

### Validation of differentially expressed genes

RNA extracted from the lung tissue was used for qRT-PCR assays. qRT-PCR was performed in duplicate using the SuperScript™ III Reverse Transcriptase (Invitrogen™) kit and iQ™ SYBR® Green Supermix as described above. qRT-PCR primers were designed for 3 randomly selected up-regulated genes based on de novo assembly and differential expression analysis (*Shisp_DN132151_c0_g1*, *Shisp_DN12103_c7_g1*, *Sfulv_DN158_c1_g1*). Primers were designed using Primer3Plus^82^ and are reported in Supplementary Table 2. No-template-controls were run on each plate. Technical replicate CT values were averaged for each gene and normalized to β-actin housekeeping gene. Results were calculated as fold change induction over uninfected lungs using the 2^−ΔΔCT^ method^74^. Additionally, to further confirm differential expression of genes, we compared the results of our current study to our previous annotation of the *S. hispidus* lung transcriptome upon RSV infection^27^ using the same DESeq2 significance parameters (*p* < 0.05, q < 0.05, l2fc >|1.0|).

### Ethics approval and consent to participate

No human subjects participated in this study. All animal procedures followed NIH and USDA guidelines and were approved by the Sigmovir Biosystems, Inc. IACUC.

## Supplementary Information

Supplementary Information 1.

Supplementary Information 2.

Supplementary Information 3.

Supplementary Information 4.

Supplementary Information 5.

Supplementary Information 6.

Supplementary Information 7.

Supplementary Information 8.

## Data Availability

The sequencing data will be deposited at the NCBI Short Read Archive (SRA) upon manuscript acceptance under BioProject PRJNA816878. For other details, please contact corresponding authors for specific data requests.
