# Supplementary material for: Species-specific transcriptomic changes upon respiratory syncytial virus infection in cotton rats
Source: Sci Rep. 2022 Oct 4;12:16579. doi: 10.1038/s41598-022-19810-4 (PMC9531660; doi:10.1038/s41598-022-19810-4)
Supplement: Supplementary file 8 — Supplementary Information 8. [file 41598_2022_19810_MOESM8_ESM.docx]

**Supplementary Data: Species-specific transcriptomic changes upon Respiratory Syncytial Virus infection in cotton rats**

Britton A. Strickland^1^, Seesandra V. Rajagopala^2^, Arash Kamali^3^, Meghan H Shilts^2^, Suman B. Pakala^2^, Marina S. Boukhvalova^3^, Shibu Yooseph^4^, Jorge C. G. Blanco^3#^, and Suman R. Das^1,2#­­^

^1^Department of Pathology Microbiology and Immunology, Vanderbilt University Medical Center, Nashville, Tennessee, USA

^2^ Department of Medicine, Vanderbilt University Medical Center, Nashville, Tennessee, USA

^3^ Sigmovir Biosystems Inc., Rockville, Maryland, USA

^4^Department of Computer Science, Genomics and Bioinformatics Cluster, University of Central Florida, Orlando, Florida, USA

**SUPPLEMENTARY FIGURES**

**Supplemental Figure 1.** (A) Top Signaling and Membrane Transport and (B) Viral and Bacterial Infection Pathways on the x-axis with total number of protein-coding genes on the y-axis. Infections in which there is published literature using cotton rats as a model are *bold italics*. Pathways assigned using TransDecoder-determined CDS followed by GhostKOALA (<https://www.kegg.jp/ghostkoala/>).

**A.**

**B.**

**Supplemental Figure 2.** Principle component analysis of RSV-infected lungs vs uninfected controls.

**
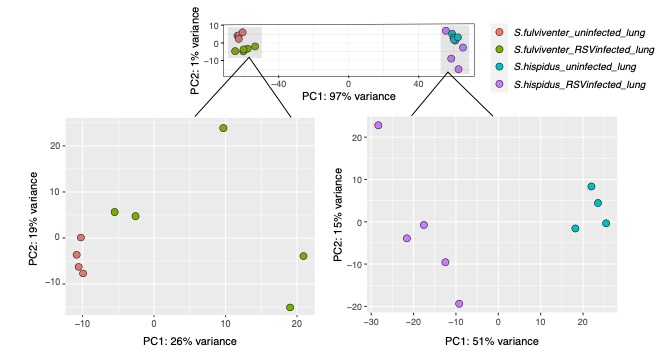
**

**Supplementary Table 1.** (A) Sequencing statistics as raw data and Trimmomatic-processed paired-end reads separated by organ type. (B) Filtering statistics for final assembly. Contigs <200bp removed using seqkit. Contigs with taxonomic annotation (BlastX) as “virus”, “bacteria”, or “fungi” were removed from transcriptome assembly. Contigs with 95% similarity were removed with CD-HIT. Primary/non-redundant mRNAs were picked by EvidentialGene tr2aacds pipeline for final assembly file.

| **Species** | **Sample Type (n)** | | **Raw Data (Mb)** | **Trimmed/QC PE Data (Mb)** | **GC%** |
| --- | --- | --- | --- | --- | --- |
|  | *Lung (4 healthy, 5 infected)* | | 410.0 | 206.0 | 53.3 |
|  | *Intestines (2)* | | 138.0 | 72.3 | 52.5 |
| *S. fulviventer* | *Heart (2)* | | 132.8 | 70.5 | 56.0 |
|  | *Spleen (2)* | | 113.8 | 56.7 | 58.5 |
|  | *Kidney (2)* | | 112.9 | 50.5 | 54.0 |
|  |  | | 907.5 | 456 |  |
| *S. hispidus* | *Lung (4 healthy, 5 infected)* | | 421.8 | 211.9 | 52.0 |
|  | *Intestines (2)* | | 127.7 | 66.4 | 54.0 |
|  | *Heart (2)* | | 109.6 | 56.4 | 50.0 |
|  | *Spleen (2)* | | 135.9 | 66.4 | 54.5 |
|  | *Kidney (2)* | | 123.8 | 63.9 | 55.0 |
|  |  | | 918.8 | 465 |  |
| **Species** | **Raw Assembly** | **Filter 200bp** | | **CD-Hit 95%** | **Evidential**  **Gene** |
| *S. fulviventer* | 1,399,089 | 1,326,282 | | 1,323,179 | 620,569 |
| *S. hispidus* | 1,399,138 | 1,326,220 | | 1,249,645 | 592,099 |

**Supplementary Table 2.** RT-qPCR validation of differentially expressed genes and primer sequences.

| **GeneID** | **Annotation** | **DESeq2 FC** | **qRT-PCR FC** | **Forward Primer** | **Reverse Primer** |
| --- | --- | --- | --- | --- | --- |
| *Shisp_DN132151_c0_g1* | *IIGP1_S.hispidus* | 1.59 | 2.28 | ACAGCTGGTCCGGATTTGAG | CCTTGCCAAAGCCATCAGC |
| *Shisp_DN12103_c7_g1* | *I27L2_S.hispidus* | 6.20 | 2.19 | ACTGTGTGCTAGCCAACCTC | CACTGACGCCAGAGAACACT |
| *Sfulv_DN158_c1_g1* | *IIGP1_S.fulviventer* | 1.33 | 2.45 | AGTCCTCAGCCAGACTCTGT | TGCCAAAGCCATCAGCATGA |
| *reference gene* | *B-actin* | – | – | GGCCAACCGTGAAAAGATGACTC | GTCCGCCTAGAAGCATTTGCG |

**SUPPLEMENTARY FILES**

**Supplementary File 1**: Reference Transcriptome (fasta) for *S. hispidus*

**Supplementary File 2**: Reference Transcriptome (fasta) for *S. fulviventer*

**Supplementary File 3**: Full annotation file for both *Sigmodon hispidus* and *S. fulviventer* (separated by xlsx sheet).

**Supplementary File 4**: KEGG annotations, Gene Ontologies, and taxonomic source of transcript annotation. All KEGG pathways can be reconstructed using data from the “KEGG_reconstruct” tab.

**Supplementary File 5**: Differential expression analysis data for both *S. hispidus* and *S. fulviventer*, including analysis by DESeq2 (including comparisons to Rajagopala et al, 2018) and GOSeq. Results are reported in separate tabs for each species.

**Supplementary File 6**: TransDecoder output identifying coding regions in each transcript for both *S. hispidus* and *S. fulviventer*.

**Supplementary File 7**: Raw expression count data (generated via Salmon; used for DESeq2 analysis) from tissues of *S. hispidus* and *S. fulviventer*.
